# Time delays in the response to the *Neisseria meningitidis* serogroup C outbreak in Nigeria – 2017

**DOI:** 10.1371/journal.pone.0199257

**Published:** 2018-06-19

**Authors:** Assad Hassan, G. U. Mustapha, Bola B. Lawal, Aliyu M. Na’uzo, Raji Ismail, Eteng Womi-Eteng Oboma, Oyeronke Oyebanji, Jeremiah Agenyi, Chima Thomas, Muhammad Shakir Balogun, Mahmood M. Dalhat, Patrick Nguku, Chikwe Ihekweazu

**Affiliations:** 1 Nigeria Field Epidemiology and Laboratory Training Programme, Abuja, Nigeria; 2 Nigeria Centre for Disease Control, Abuja, Nigeria; 3 World Health Organisation, Borno, Nigeria; University of Cambridge, UNITED KINGDOM

## Abstract

**Background:**

Nigeria reports high rates of mortality linked with recurring meningococcal meningitis outbreaks within the African meningitis belt. Few studies have thoroughly described the response to these outbreaks to provide strong and actionable public health messages. We describe how time delays affected the response to the 2016/2017 meningococcal meningitis outbreak in Nigeria.

**Methods:**

Using data from Nigeria Centre for Disease Control (NCDC), National Primary Health Care Development Agency (NPHCDA), World Health Organisation (WHO), and situation reports of rapid response teams, we calculated attack and death rates of reported suspected meningococcal meningitis cases per week in Zamfara, Sokoto and Yobe states respectively, between epidemiological week 49 in 2016 and epidemiological week 25 in 2017. We identified when alert and epidemic thresholds were crossed and determined when the outbreak was detected and notified in each state. We examined response activities to the outbreak.

**Results:**

There were 12,535 suspected meningococcal meningitis cases and 877 deaths (CFR: 7.0%) in the three states. It took an average time of three weeks before the outbreaks were detected and notified to NCDC. Four weeks after receiving notification, an integrated response coordinating centre was set up by NCDC and requests for vaccines were sent to International Coordinating Group (ICG) on vaccine provision. While it took ICG one week to approve the requests, it took an average of two weeks for approximately 41% of requested vaccines to arrive. On the average, it took nine weeks from the date the epidemic threshold was crossed to commencement of reactive vaccination in the three states.

**Conclusion:**

There were delays in detection and notification of the outbreak, in coordinating response activities, in requesting for vaccines and their arrival from ICG, and in initiating reactive vaccination. Reducing these delays in future outbreaks could help decrease the morbidity and mortality linked with meningococcal meningitis outbreaks.

## Introduction

Meningococcal meningitis outbreaks in Africa are frequently detected too late to enable appropriate control and preventive actions to limit their impact [1]. High mortality rates often characterise the onset of outbreaks before appropriate measures are taken [2], leading to high attack rates of up to 100 to 800 per 100,000 populations, and case fatality ratios (CFR) of between 5 and 10% [3]. The age group mostly affected by outbreaks of meningococcal meningitis are the 5–15 year olds [4], and about 10–20% of patients develop neurological sequalae such as deafness, learning disabilities and epilepsy [5]. These figures are likely to be higher due to the sub-optimal reporting system to record cases [3, 6]. Given that outbreaks occur frequently in the 26 contiguous countries that make up the African meningitis belt [7], questions have been asked on why outbreaks cannot be detected earlier to enable a more rapid public health response.

Between January and June 1996, the largest ever epidemic of meningococcal meningitis in Nigeria affected a reported 109,580 cases leading to 11,717 deaths (CFR 10.7%) [8]. A review of health facility records at that time revealed a rise in incidence of meningitis cases from October, 1995 with the epidemic threshold being crossed in November 1995 [6]. Yet, a strong response did not start until February 1996. In 2009, another epidemic associated with late case detection and case management saw a reported 51,792 cases and 2,364 deaths (CFR 4.6%) [9]. A similar outbreak in 2015 with CFR of 5% showed evidence of late detection and reporting of cases [10]. In each of the afore-mentioned outbreaks, inadequate surveillance led to delays in the response.

Early warning systems are put in place to ensure timeliness of detection and to potentially reduce morbidity and mortality [11, 12]. Two epidemiological intervention thresholds for meningitis outbreaks—alert and epidemic thresholds—have been agreed to guide timely implementation of response activities to meningococcal outbreaks [2]. The doctrine of the meningitis alert and epidemic thresholds is to immediately commence control activities once these thresholds have been crossed and the serogroup responsible identified. The recommendation is to conduct a reactive vaccination campaign within four weeks of crossing the epidemic threshold in both the population affected and adjacent populations considered to be at risk [2, 13]. The International Coordinating Group (ICG) on Vaccine Provision was set up to improve the availability of vaccines to respond to epidemics. It was formed in 1997 to respond to meningitis outbreaks through provision of vaccines in a coordinated, equitable, and timely manner [8]. The ICG recommends that a request for vaccines from an affected country should be sent to the ICG immediately the epidemic threshold has been crossed. Vaccines are expected to arrive in country 10 days after such a request is submitted [8]. It is also recommended that a Rapid Response Team (RRT) from the central coordinating level should be deployed to the affected areas to support surveillance (data collection, analysis and transmission) and other outbreak response activities [9].

Between November 22, 2016 and June 23, 2017, there was a large outbreak of meningococcal meningitis due to *Neisseria meningitidis* serogroup C (NmC) in northern Nigeria which mostly affected the states of Zamfara, Sokoto and Yobe. We describe time delays in different stages of the outbreak response, factors that might have been responsible, and recommend measures to reduce these delays in the future.

## Methods

For Zamfara, Sokoto and Yobe states, case counts and deaths were determined against time-lines for surveillance, treatment and care; and reactive vaccination in response to the outbreak through the following activities:

### Surveillance data assessment

Data from the weekly incidence of suspected meningococcal meningitis cases and deaths for Zamfara, Sokoto and Yobe states from epidemiological week 49 in 2016 to epidemiological week 25 in 2017 from the electronic database of the Nigeria Centre for Disease Control (NCDC) were used to determine the number of cases by ward and local government area (LGA) for each of the three states respectively. For each state, we calculated the weekly attack and death rates by LGA. We then identified when the alert and epidemic thresholds were crossed by each of the affected LGAs and determined when the outbreak was detected and notified in each state to NCDC. We also calculated case-fatality ratios (CFR) for each of the states.

### Reactive vaccination data assessment

Data on reactive vaccination was obtained from records of the NCDC, National Primary Healthcare Development Agency (NPHCDA), World Health Organisation (WHO), field situation reports by RRTs deployed from NCDC to Zamfara, Sokoto and Yobe states and also from the respective State Epidemiologists, State Disease Surveillance and Notification Officers (SDSNOs); and State Immunization Officers (SIOs). We assessed request letters for vaccines against *Neisseria meningitidis* serogroup C written by Nigeria to the International Coordinating Group (ICG) on vaccines. These data were used to determine when requests for the vaccines were sent and when the vaccines arrived in-country. We determined the quantity of vaccines requested against the quantity received and calculated proportion of received vaccines and the time it took to initiate reactive vaccination campaigns in each of the three states.

The National Code of Health Research Ethics of the National Health Research Ethics Committee of Nigeria exempted this study from ethical committee oversight. All data used in this study were fully anonymized, that is, cleared of information that could be used to identify individual patients/cases, prior to access by any of the authors.

## Results

### Zamfara state

In epidemiological week 47 of 2016, there was an unknown febrile illness that had already affected over 444 cases with 45 deaths across Birnin Magaji, Maradun and Zurmi local government areas (LGAs). Health workers in these LGAs had been managing the cases for severe malaria unsuccessfully, many of whom tested negative for malaria. In epidemiological week 2 of 2017, laboratory results of cerebrospinal fluid (CSF) samples taken from affected individuals returned positive for *Neisseria meningitidis* serogroup C and negative for viral haemorrhagic fevers. The first laboratory confirmed meningococcal meningitis case was in Zurmi LGA in epidemiological week 2 of 2017.

**Fig 1:**
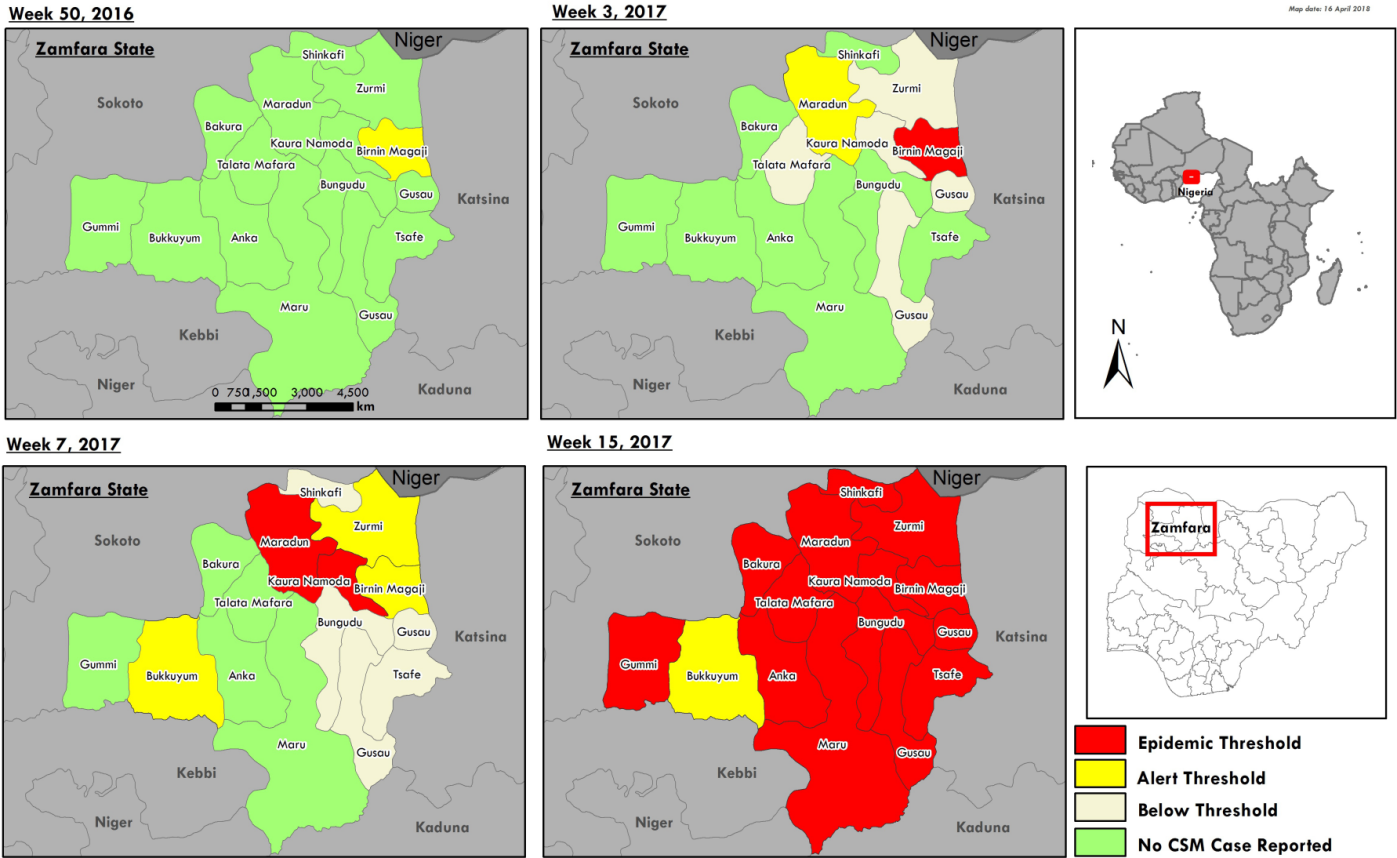
Geographical progression of cerebrospinal meningitis (CSM) outbreak in Zamfara.

Based on above confirmation of *Neisseria meningitidis*, a retrospective re-classification of the unknown febrile illness cases was done and consequently, Birnin Magaji and Maradun LGAs crossed the alert threshold in epidemiological week 50 of 2016 and week 2 of 2017 respectively. Birnin Magaji crossed the epidemic threshold in epidemiological week 3, 2017 (Fig 1). This was followed by Kaura Namoda and Zurmi LGAs who crossed the alert threshold in epidemiological weeks 4 and 5 respectively. However, the outbreak was notified and a line list was only submitted to the NCDC in epidemiological week 6. During epidemiological week 7, Maradun and Kaura Namoda LGAs crossed the epidemic threshold. The first formal meeting of the State Emergency and Preparedness Response Committee, which set up a State RRT with terms of reference to respond to the outbreak, held in epidemiological week 8, i.e. five weeks after the epidemic threshold was crossed. In epidemiological weeks 9 and 11, Shinkafi and Zurmi LGAs crossed the epidemic threshold for each week respectively. Between epidemiological weeks 12 and 15, eight more LGAs had crossed the epidemic threshold. Out of 14 LGAs in Zamfara state, 13 crossed the epidemic threshold while one crossed only the alert threshold during the outbreak.

The NCDC did not have a protocol for activating an emergency operations centre (EOC) for meningitis outbreaks. As it became clear that the outbreak was spreading beyond Zamfara state, a meeting of NCDC and other stakeholders saw the need to activate an EOC for the response. As the outbreak escalated, in epidemiological week 14, a national cerebrospinal meningitis (CSM) r EOC was activated at NCDC on April 3 and a national RRT was deployed to Zamfara. National case management teams (NCMT) were deployed from NCDC on May 6 in epidemiological week 18. New leadership at the Zamfara State Epidemiology Unit in late 2016 (both the State Epidemiologist and SDSNO were newly appointed) i.e. during onset of the unknown febrile illnesses, inadequate surveillance capacity at the LGA level, logistical challenges in accessing hard to reach areas (lack of operational vehicles), inadequate skilled clinicians to perform lumbar punctures, inadequate trans-isolate media to transport cerebrospinal fluid (CSF) samples to the laboratory and inadequate laboratory consumables were captured in situation reports by RRTs as challenges that were faced in responding to the outbreak.

Four ICG requests were sent from Nigeria during the outbreak period and less than half (approximately 41%) of the number of requested vaccines arrived in-country. Sub-optimal coordination between NCDC, NPHCDA, the states, and other stakeholders in developing and sending requests to ICG for reactive vaccination was recorded at the national CSM EOC. Feedback from ICG to NPHCDA was that the total number of vaccines requested by the three states exceeded global *Neisseria meningitidis* serogroup *C* vaccine stockpile.

**Fig 2:**
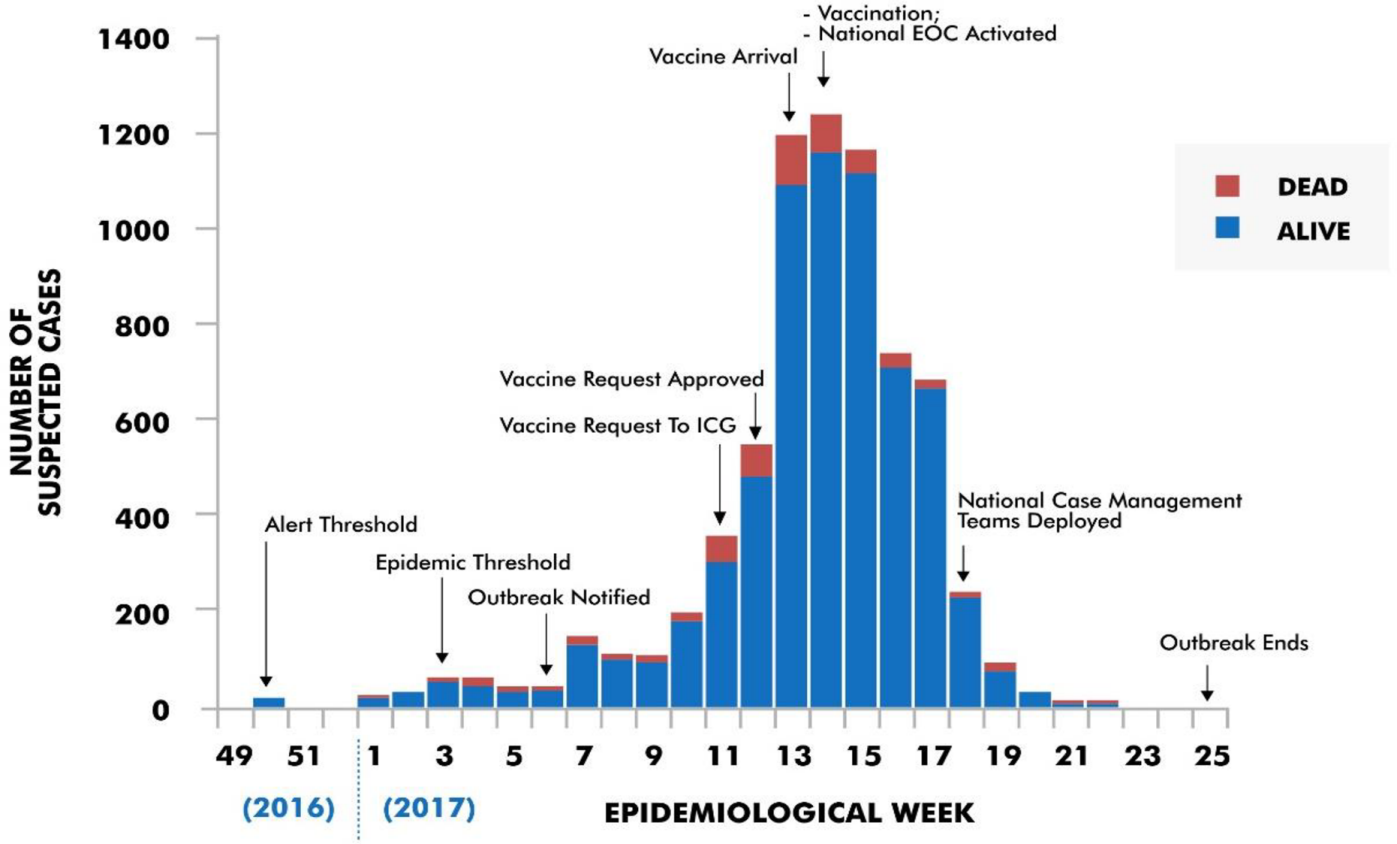
Reported cases of meningococcal meningitis in Zamfara between epidemiological weeks 49, 2016 and 25, 2017.

A request for 3,617,241 doses of conjugate meningitis vaccine (A+C) was sent to the ICG on March 13, 2017 (epidemiological week 11) and approval was obtained on March 20, 2017 (epidemiological week 12). Of the 3,617,241 doses of vaccines requested, 420,000 arrived in Nigeria on March 27 and subsequently Zamfara on March 30, 2017 (both in epidemiological week 13) respectively. The first phase of reactive vaccination of eligible persons between 1 to 29 years of age in affected wards held between April 5 and 9, 2017 (epidemiological week 14) giving a total of three weeks from vaccine request to vaccination campaign (Fig 2). A second batch of 144,000 doses of AC and 587,980 doses of ACWY vaccines from ICG arrived Zamfara on May 19, 2017 (epidemiological week 20) and second phase of vaccination was carried out between May 22 and 26, 2017 (epidemiological week 21).

A case count of 7,140 and 553 deaths (CFR: 7.7%) were recorded between epidemiological weeks 49 of 2016 and 25 of 2017. Of the 553 deaths, 46 (8.3%) occurred before the outbreak was notified to NCDC, 382 (69.1%, cumulative 77.4%) occurred between notification and commencement of vaccination, 80 (14.5%, cumulative 91.9%) occurred between commencement of vaccination and national EOC activation, 20 (3.6%, cumulative 95.5%) occurred between national EOC activation and deployment of NCMT, and 25 (4.5%, cumulative 100%) occurred between deployment of NCMT and end of the outbreak.

### Sokoto state

**Fig 3:**
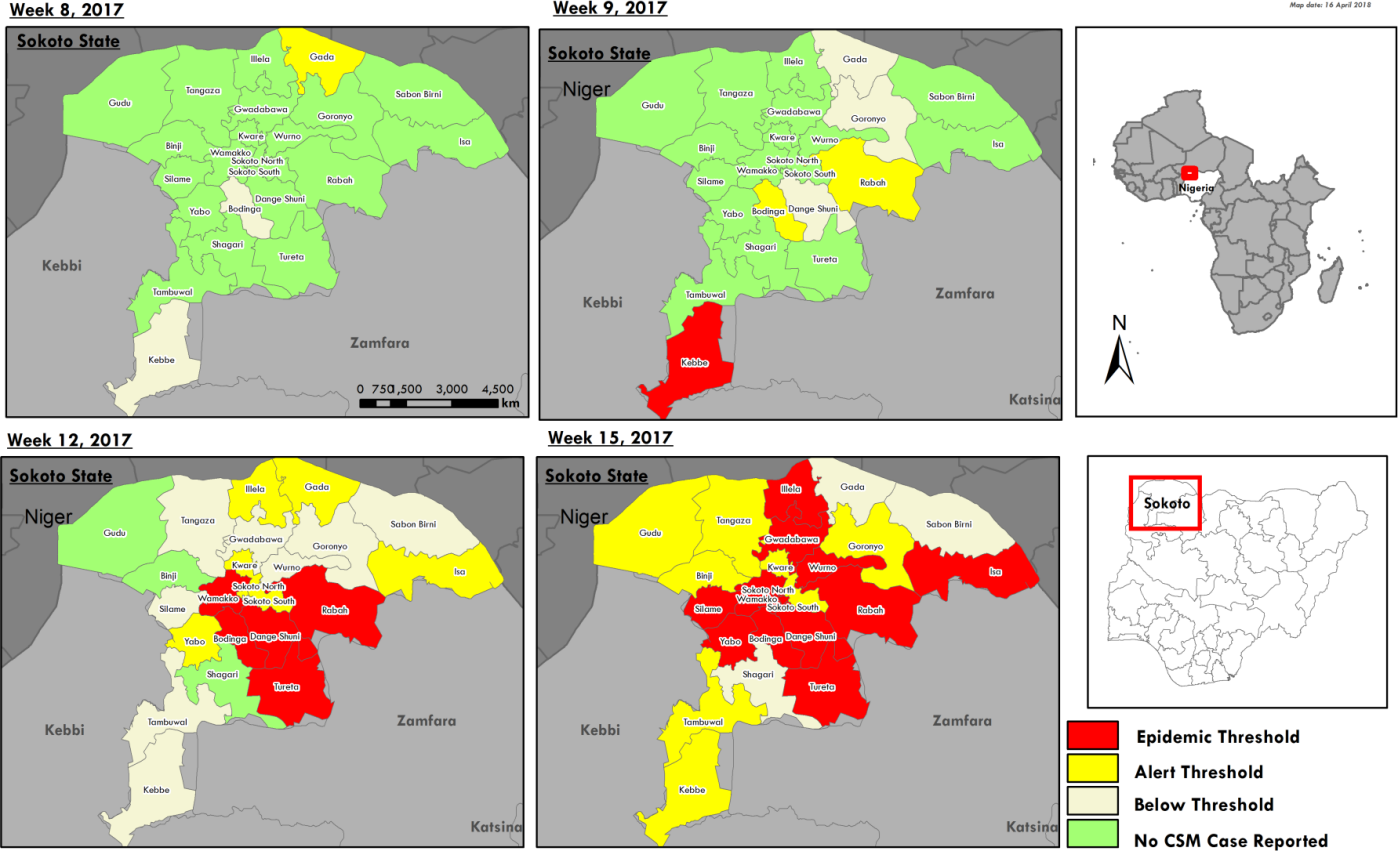
Geographic progression of cerebrospinal meningitis (CSM) outbreak in Sokoto.

In Sokoto State, Gada was the first LGA to cross the alert threshold in epidemiological week 8 and was followed by Bodinga and Rabah LGAs in epidemiological week 9 respectively. Kebbe LGA crossed epidemic threshold in epidemiological week 9 (Fig 3) and the outbreak was notified in epidemiological week 12. The outbreak spread rapidly as 19 of the 23 LGAs in the state crossed the epidemic threshold while four crossed only the alert threshold by epidemiological week 18.

In epidemiological week 14, a national RRT was deployed to Sokoto. NCMT were deployed to support case management from NCDC in epidemiological week 18. RRT situation reports indicated that the state did not notify NCDC of the outbreak earlier than epidemiological week 12 because the state assumed it would be able to adequately respond to the outbreak. Poor data management at health facilities and at the State Epidemiology Unit; insufficient number of trained health care workers to carry out lumbar punctures; lack of trans-isolate media to transport collected CSF, and irregular distribution of drugs across treatment camps were also among the challenges captured by the RRTs.

**Fig 4:**
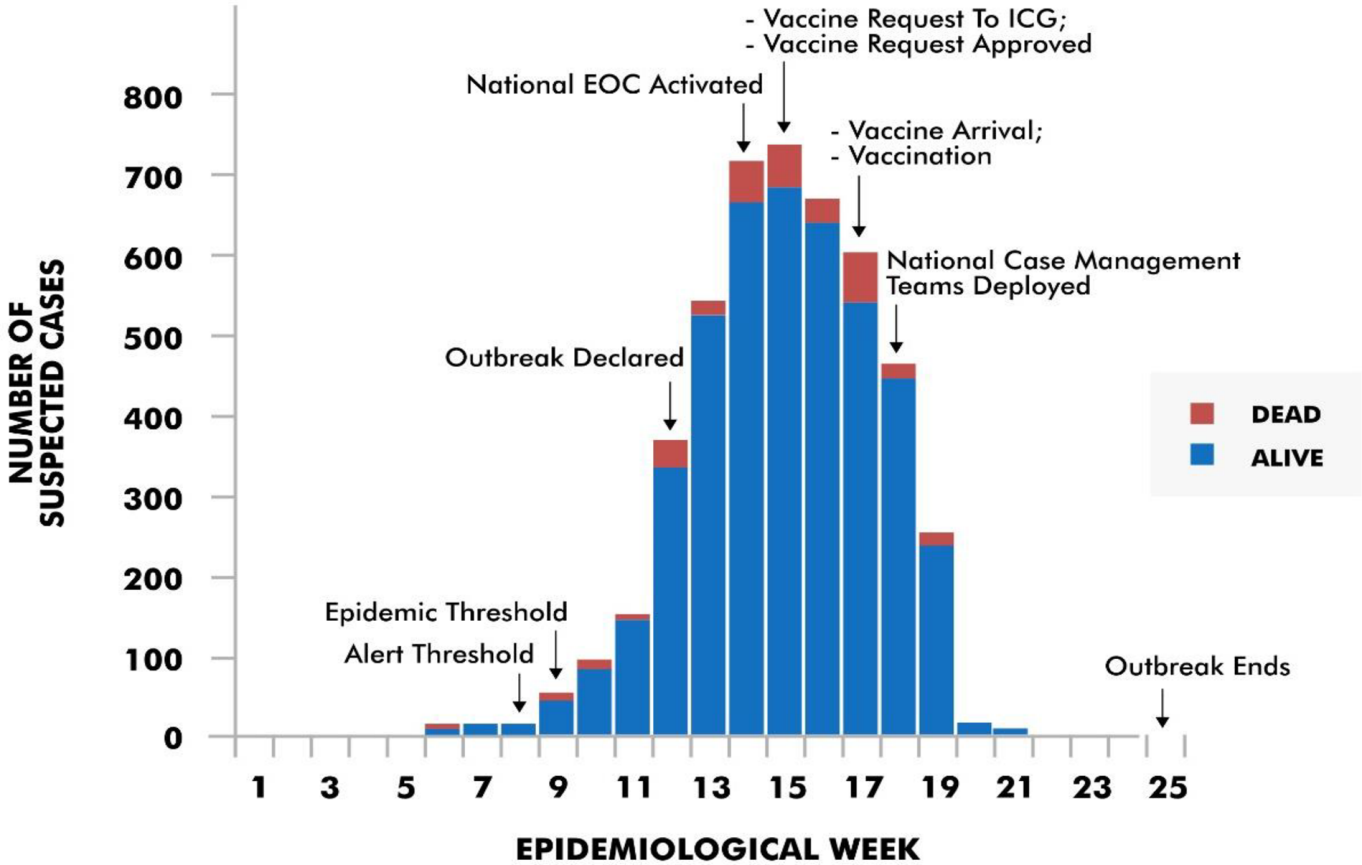
Reported cases of meningococcal meningitis in Sokoto between epidemiological weeks 1 and 25, 2017.

A request for 823,970 doses of C conjugate vaccines was submitted to the ICG on April 10, 2017 and approval obtained on April 11, 2017 (both in epidemiological week 15) for vaccination in only nine out of the 24 LGAs in the state. The first batch of 97,920 doses arrived Sokoto on April 24, 2017 (epidemiological week 17). The vaccination campaign was delayed by one day because the vaccines were not bundled with needles to administer the vaccines. On April 28, the vaccination campaign kicked off and ended on May 2, 2017 (between epidemiological weeks 17 and 18) (Fig 4). The second batch of vaccines arrived on April 25 and vaccination started on May 4 and ended on May 6, 2017 (epidemiological week 18). The remaining vaccines arrived in three batches between April 26 and May 1, 2017 respectively.

A case count of 4,980 and 283 deaths (CFR: 5.7%) were recorded between epidemiological weeks 2 and 25 of 2017. Of the 283 deaths, 50 (17.7%) occurred before the outbreak was declared, 73 (25.8%, cumulative 43.5%) occurred between outbreak declaration and national EOC activation, 132 (46.6%, cumulative 90.1%) occurred between national EOC activation and commencement of vaccination, 15 (5.3%, cumulative 95.4%) occurred between commencement of vaccination and deployment of NCMT, and 13 (4.6%, cumulative 100%) occurred between deployment of NCMT and end of the outbreak.

### Yobe state

**Fig 5:**
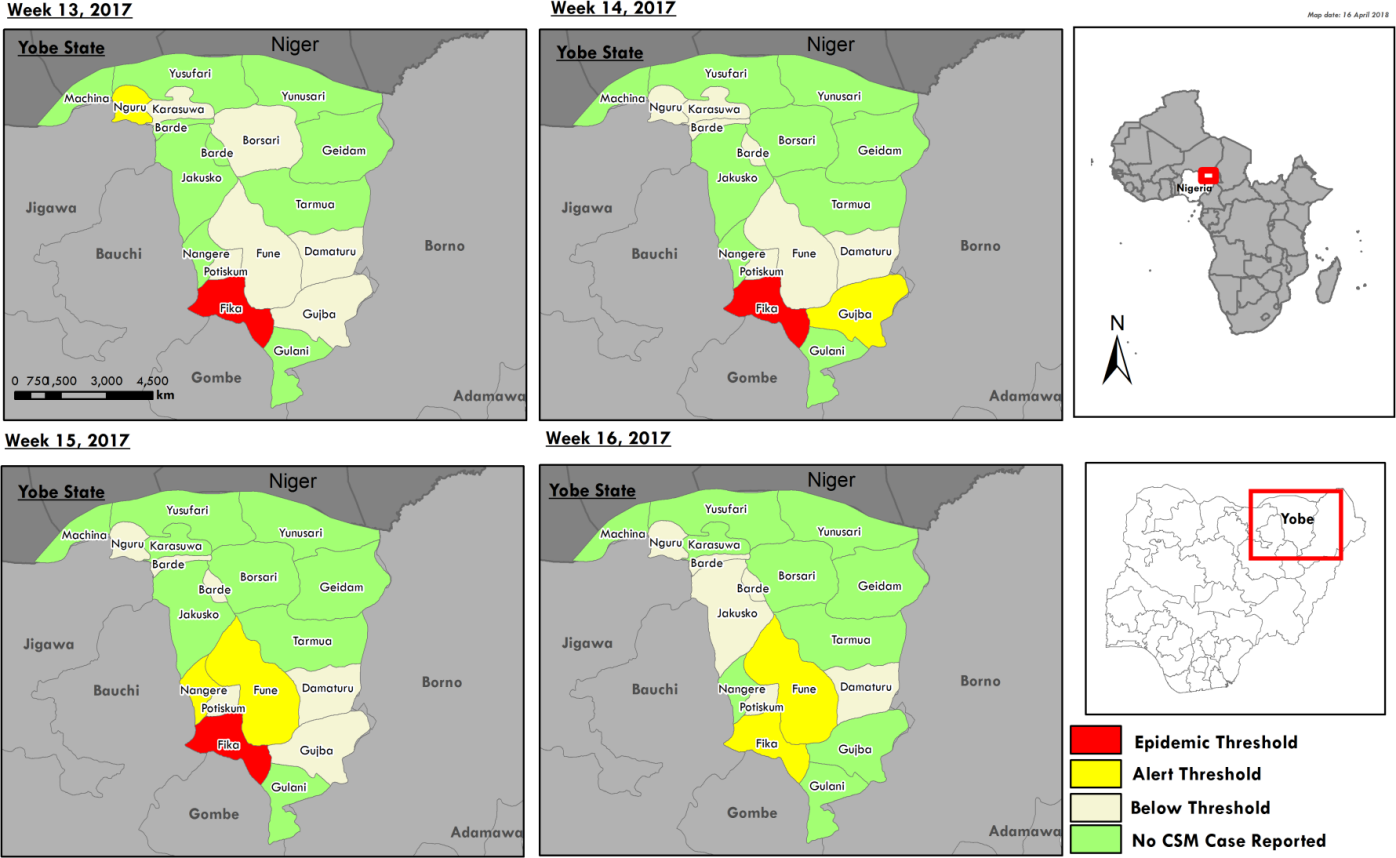
Geographic progression of cerebrospinal meningitis (CSM) outbreak in Yobe.

Between epidemiological weeks 13 and 17, four LGAs had crossed the alert threshold. In epidemiological week 13, only Fika LGA had crossed the epidemic threshold (Fig 5). Out of 17 LGAs in the state, only Fika LGA crossed the epidemic threshold while four crossed only the alert threshold.

A national RRT was deployed in epidemiological week 17. A second team was deployed to support case and data management in epidemiological week 21. Insufficient number of health workers and weak laboratory capacity were recorded as challenges that were faced in responding to the outbreak by the RRTs.

**Fig 6:**
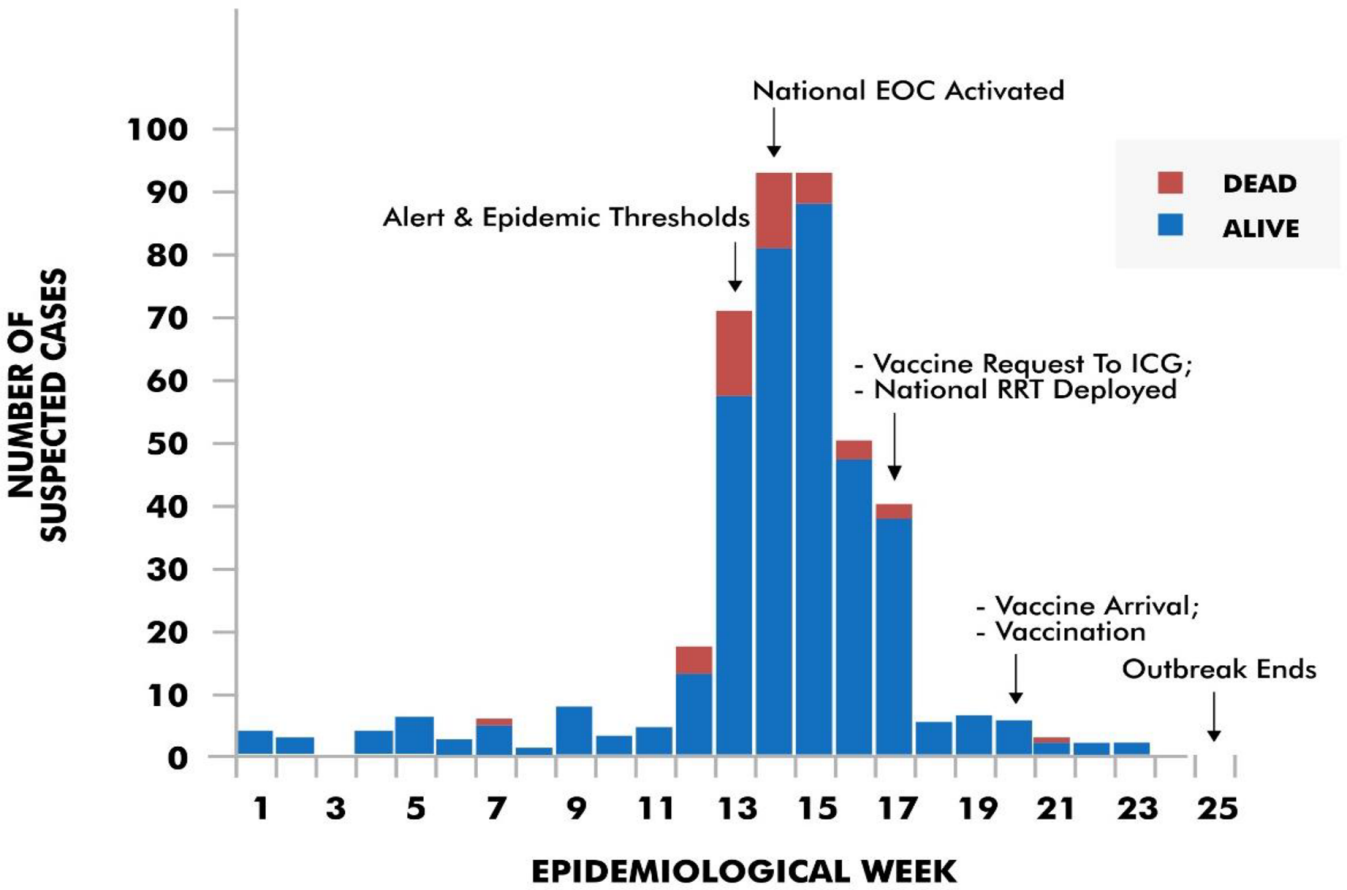
Reported cases of meningococcal meningitis in Yobe between epidemiological weeks 1 and 25, 2017.

Request letter for 869,089 doses of vaccines was sent to ICG in epidemiological week 17 (Fig 6). About 189,280 doses of Men ACW PS vaccines arrived on May 17, 2017 and reactive vaccination commenced on May 19, 2017 (both in epidemiological week 20 respectively).

A case count of 415 and 41 deaths (CFR: 10.0%) were recorded between epidemiological weeks 1 and 25 of 2017. Of the 41 deaths, 30 (73.2%) occurred before the national EOC activation, 10 (24.4%, cumulative 97.6%) occurred between national EOC activation and deployment of national RRT, and one death (2.4%, cumulative 100%) occurred between deployment of national RRT and commencement of vaccination. Outbreak ended in week 25.

**Fig 7:**
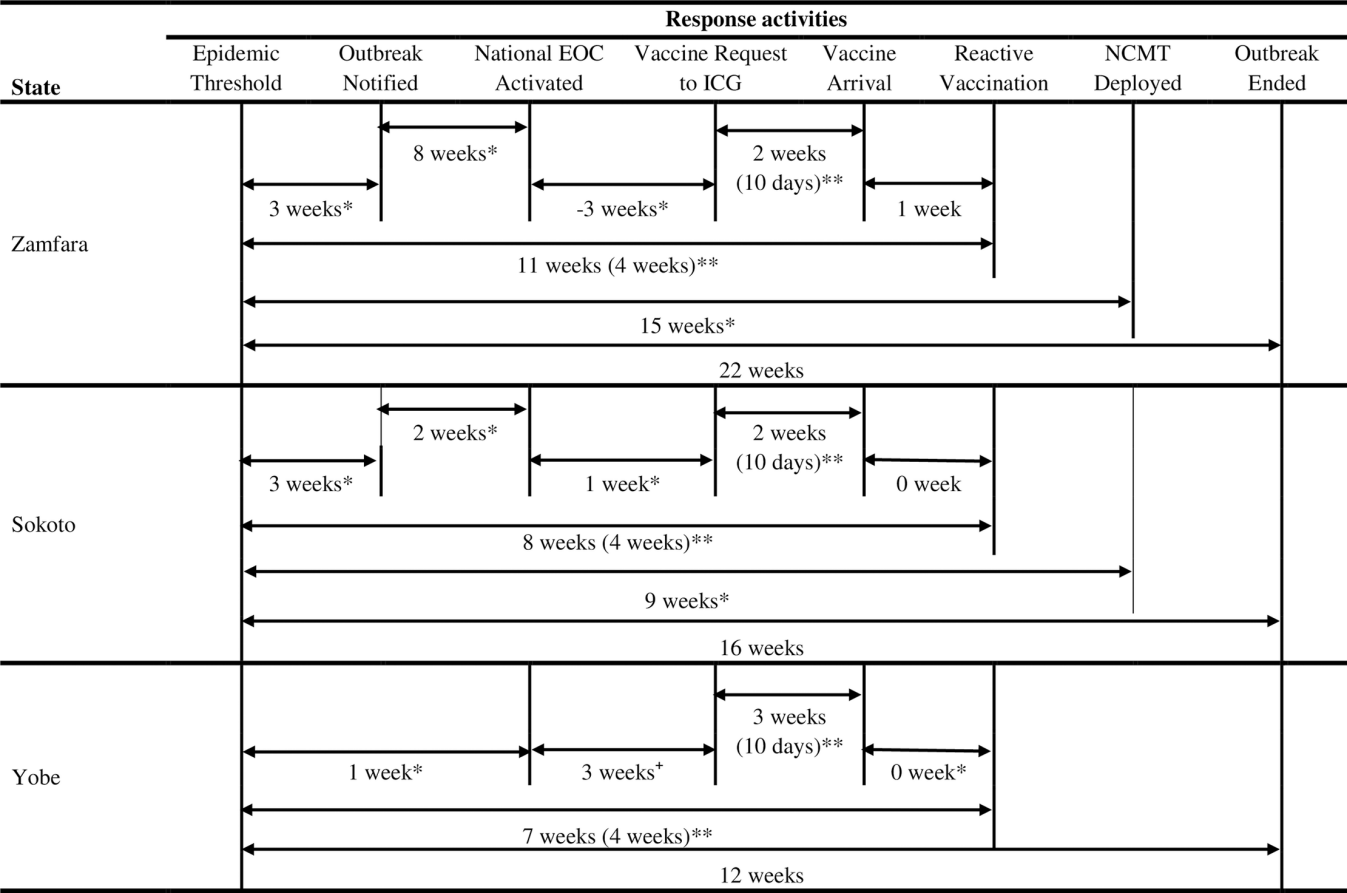
Time-line of surveillance and response activities to the outbreak of *Neisseria meningitidis* serogroup C in Nigeria, 2016/2017. ^**+**^Vaccine request to ICG preceded the National EOC activation, *Expected to be done when epidemic threshold is reached, **Expected time interval.

The population of the LGAs in the three states ranged from 89,943 to 541,825 people. Between epidemiological weeks 22 and 25 i.e. over a four week period, no LGA was in either alert or epidemic threshold in all the states. Based on this, the outbreak was declared over in week 25. Overall, the outbreak lasted for an average of 17 weeks in the three states (Fig 7).

## Discussion

This study on the response to the 2016/2017 regional outbreak of meningococcal meningitis in Sokoto, Yobe and Zamfara states has shown that there were delays at different stages in the response to the outbreak which might have aggravated this public health emergency. If these delays had been minimised, it is likely that fewer cases and deaths would have been recorded during the outbreak. We found delays in four major areas: detection and notification of the outbreak by the states, initiating an integrated national response to the outbreak by the NCDC, request for vaccines and response from ICG, and initiating reactive vaccination campaigns. We also found that various factors might have contributed to these delays.

Delays in detection and reporting is perhaps the most crucial of all the delays that were observed in our study. This is because subsequent operational interventions targeted at controlling meningitis outbreaks such as standard treatment and care, reactive vaccination, and social mobilization are all hinged on early detection and notification [1, 7, 10]. Good surveillance is critical to ensure that the first cases of an outbreak are detected on time and the serogroup of the meningococcus responsible for infection is identified [11]. Similar delays were previously reported in a study conducted by Paireau et al, which showed delayed meningococcal meningitis epidemic notifications in 15 affected districts in Niger Republic between 2003 and 2009 [12]. On the other hand, in 2015/2016, an outbreak of bacterial meningitis in Ghana was rapidly detected and notified, consequently, the etiologic agent was quickly identified which informed other targeted response actions [13]. While a case fatality ratio of 9.2% was still recorded at the end of the outbreak [14], the absolute number of deaths was probably lower than it could have been.

Transition of leadership at the Zamfara Epidemiology Unit might have been one of the factors implicated in delaying detection and reporting of cases in the state. Perhaps this finding is an indication of a situation where capacity is built around individuals instead of systems. The process of change in leadership usually presents with both challenges and opportunities. Depending on how and when a leadership transition occurs, its effects could be positive or negative. This period of change could present with vulnerable gaps which could lead to mistakes and adverse outcomes [15]. Moving forward, systems should be designed in such a way that there is little or no interruption during transitions. Furthermore, a balance should be made by coupling inexperienced new members with experienced members for optimal performance. The state government should also provide adequate resources for them to carry out their responsibilities effectively. Another factor that potentially delayed notification of the outbreaks to the NCDC by the states was their perception of the ability to contain the outbreaks without external assistance. While this may seem true and possible on the surface, more often than not, the states do not have the adequate technical and material resources to respond to meningococcal outbreaks on their own [16]. Giving credence to this statement are other findings in this study which implied that inadequate skilled personnel to carry out lumbar punctures, inadequate medical consumables and inadequate laboratory capacity might also have impacted on delayed detection of the outbreak in the three states. The spread of meningococcal meningitis epidemics can be difficult to predict. Prompt notification by states to NCDC will help ensure a positive multiplier effect as inadequacies and gaps in responding to the outbreaks will be closed within the shortest possible time. While training adequate number of physicians to carry out lumbar punctures during meningitis epidemics might be a huge undertaking and may be more of a long term plan, states should be able to maintain stockpiles of medical and laboratory consumables as part of preparedness and response plans against the almost yearly meningitis outbreaks being experienced in these states.

We found an average of nine weeks from the initial epidemic threshold being passed and the initiation of the reactive vaccination campaigns in the three states, versus a recommended duration of four weeks. Delays in requesting for vaccinations from the ICG and consequent delays in reactive vaccinations have been previously reported in other African countries. Asiedu-Bekoe et al found a delay in request for vaccines from the ICG in the 2015/2016 meningococcal outbreak in Ghana [17]. Different duration of delays in reactive vaccination have been reported by many other countries. It took two to five weeks in Sudan [1, 18], four to nine weeks in Burundi and six weeks in Chad [1] to initiate reactive vaccinations to meningitis outbreaks measured from the point the epidemic threshold was crossed. For every week of delay in reactive vaccination, there is a reduction by three to eight percent of cases that would have been prevented in a meningococcal outbreak [1], demonstrating the impact of these delays. Using data from African meningitis belt countries, Trotter et al found that 17 cases would be protected if a reactive vaccination was carried out within six weeks of crossing the epidemic threshold and 54 cases would be protected if reactive vaccination was carried out within four weeks of crossing the epidemic threshold [19]. Although it could be argued that reactive vaccination in the three states probably did not impact ending the outbreak across the three states, it is highly likely that fewer cases and deaths might have been recorded if the reactive vaccination campaigns held earlier than they did during the outbreak. The NPHCDA is the Nigerian government agency that takes the lead and responsibility for developing requests for vaccines from the ICG while the NCDC is responsible for providing epidemiological data to support such requests. These are done collaboratively with affected State Primary Health Care Development Agencies and with the corresponding State Epidemiology Units. To improve quality and timely request to ICG, a permanent technical ICG request team should be established comprising members from each of the above mentioned government institutions and any other relevant body. Additionally, the NPHCDA should retain a national stockpile of emergency meningococcal vaccines which can be used to initiate quick response while awaiting vaccines from the ICG.

Considering the usual magnitude of meningitis outbreaks in this region, a delayed integrated national response to an outbreak can have severe consequences. This is more so the case in a context like Nigeria where resources are rarely sufficient in the states or LGAs to manage the outbreaks. Coordination of response activities in resource-poor settings in Africa is vital to successful interventions. Therefore, a central response coordination structure such as an EOC should be promptly set up once an epidemic is suspected so as to eliminate duplication of response efforts and to coordinate scarce resources [11]. The consequences of delayed national response to an outbreak could lead to an increase in avoidable morbidity and mortality along with other adverse outcomes such as potential for international spread of disease and disruption in trade as demonstrated in a study by Heymann et al, who gave illustrations of these consequences as previously seen in India and the Democratic Republic of the Congo [20]. To complement the central EOC, protocols should be developed by the NCDC to guide preparedness and response against future meningococcal meningitis outbreaks. Such protocols should clearly spell out roles and responsibilities of all stakeholders in epidemic meningitis response activities.

## Conclusion

Timely detection and reporting of meningococcal outbreaks is an important control strategy. Good surveillance is the prerequisite for subsequent control measures such as timely standard treatment and care as well as reactive vaccination campaigns. Coordination of response activities also minimizes delays and wastage of scarce resources. Moving forward, improvements in response to meningococcal meningitis outbreaks should focus on reducing the delays in these four stages of response i.e. detection and notification of the outbreak by the states, initiating an integrated national response to the outbreak by the NCDC, request for vaccines and response from ICG, and initiating reactive vaccination campaigns.

## Supporting information

S1 Dataset Anonymized 2017 CSM Outbreak AR Workbook.rar. (RAR)
